# Insights into the mechanism of L-malic acid on drip loss of chicken meat under commercial conditions

**DOI:** 10.1186/s40104-023-00987-1

**Published:** 2024-01-29

**Authors:** Haijun Sun, Xue Yan, Lu Wang, Ruimin Zhu, Meixia Chen, Jingdong Yin, Xin Zhang

**Affiliations:** 1 State Key Laboratory of Animal Nutrition and Feeding, College of Animal Science and Technology, China Agricultural University, Beijing, 100193 China; 2 New Hope Liuhe Co., Ltd./Key Laboratory of Feed and Livestock and Poultry Products Quality & Safety Control, Ministry of Agriculture, Chengdu, Sichuan 610023 China; 3 Institute of Animal Husbandry and Veterinary Medicine, Beijing Academy of Agriculture and Forestry Sciences, Beijing, 100097 China

**Keywords:** Drip loss, Immune response, L-malic acid, Meat quality, Metabolome, Transcriptome

## Abstract

**Background:**

A deterioration in the meat quality of broilers has attracted much more attention in recent years. L-malic acid (MA) is evidenced to decrease meat drip loss in broilers, but the underlying molecular mechanisms are still unclear. It’s also not sure whether the outputs obtained under experimental conditions can be obtained in a commercial condition. Here, we investigated the effects and mechanisms of dietary MA supplementation on chicken meat drip loss at large-scale rearing.

**Results:**

Results showed that the growth performance and drip loss were improved by MA supplementation. Meat metabolome revealed that L-2-aminoadipic acid, β-aminoisobutyric acid, eicosapentaenoic acid, and nicotinamide, as well as amino acid metabolism pathways connected to the improvements of meat quality by MA addition. The transcriptome analysis further indicated that the effect of MA on drip loss was also related to the proper immune response, evidenced by the enhanced B cell receptor signaling pathway, NF-κB signaling pathway, TNF signaling pathway, and IL-17 signaling pathway.

**Conclusions:**

We provided evidence that MA decreased chicken meat drip loss under commercial conditions. Metabolome and transcriptome revealed a comprehensive understanding of the underlying mechanisms. Together, MA could be used as a promising dietary supplement for enhancing the water-holding capacity of chicken meat.

## Background

Chicken meat is a commonly accepted source of high-quality animal protein by consumers due to its highly digestible proteins and low-fat contents. Recently, to increase the growth performance, high-density feeding patterns before slaughter have made broilers more susceptible to oxidative stress, resulting in a significant increase in the occurrence of pale, soft, and exudative (PSE)-like chicken meat [1]. Alarmingly, this condition accounts for more than 20% of the total production in commercial chicken slaughter plants in many countries [2, 3]. Poor water-holding capacity (WHC) is a characteristic of PSE meat [4], which has a direct bearing on the color and tenderness of meat [5]. Additionally, WHC influences consumer acceptance, the final weight of product, and shelf life [6]. Poor WHC also leads to the loss of certain vitamins, minerals, salts, and protein, which diminishes the nutritive value [7]. Therefore, gaining a better understanding of the molecular background and establishing strategies to improve WHC in chickens is very important.

Drip loss is a key parameter of WHC in fresh meat. As reviewed previously, the rate and extent of pH decline are closely related to drip loss during the conversion of muscle to meat [5]. When the pH of the muscle reaches the isoelectric point, an overall reduction of reactive groups available for water binding on muscle proteins occurs. Lower ultimate pH is closely associated with higher glycolytic potential [8]. Metabolomic analysis also indicated the differences in carbohydrate metabolism, adenine and adenosine salvage, adenosine nucleotides degradation, arsenate detoxification, and methylglyoxal degradation between chicken breast muscle with higher and lower drip loss [9]. Dietary nutrients have a significant impact on chicken meat quality attributes, including shear force, pH value, and taste [10, 11]. Antioxidants, such as pterostilbene, rutin, and betaine, have been used to decrease drip loss by improving the antioxidant capacity of broilers [12–14].

L-malic acid (MA) is naturally occurring in immature fruits, including hawthorns, apples, and grapes, processing a variety of biological activities including anti-oxidative and anti-bacterial [15, 16]. Due to its small contents in fruits, MA is mainly produced through enzymatic synthesis and microbial fermentation [17]. As reported in our previous study, dietary supplementation of MA attenuated oxidative stress and inflammation in weaned piglets [18]. Importantly, MA administration increased the oxidative fiber percentage and decreased the drip loss of pork [18]. Meanwhile, the beneficial effects of MA on drip loss were also observed using 288 Arbor Acres (AA) broilers [19], however, the exact mechanism is unknown. It’s also not sure whether the outputs obtained under experimental conditions could translate into commercial conditions.

In the present study, we hypothesized that dietary MA supplementation would modify the metabolism in the skeletal muscle, subsequently contributing to the improved WHC of chicken meat under commercial conditions. Therefore, this research was performed under commercial conditions to assess the effects of MA administration on chicken meat drip loss. Moreover, MS-based metabolomics and transcriptomics were applied to reveal the mechanism underlying the beneficial effects of MA on drip loss.

## Materials and methods

### Animals, diets, and experimental design

A total of 96,000 one-day-old AA broilers (half male and half female) were weighed and randomly assigned into two groups with 64,000 broilers for the control group and 32,000 broilers for the MA group. There were 1,000 cages and 500 cages in the control and MA group, with 60–70 broilers per cage. Broilers were fed at a commercial farm (New Hope Liuhe, Shandong, China) for a 38-d experiment. The experimental trial was divided into 3 stages: d 0–13 (starter period), d 14–20 (grower period), and d 21–38 (finisher period). The control group was fed the corn-soybean meal basal diet containing 0.8% zeolite power (carrier of MA), and the MA group received the basal diet containing 0.8% MA complex. The basal diet was formulated to meet the nutrition requirements of the Chinese Feeding Standard of chicken [20]. MA complex was obtained from Anhui Sealong Biotechnology Co., Ltd. (Bengbu, Anhui Province, China), which was composed of 20% MA and 80% carrier (zeolite power). Ingredients and nutrient compositions of basal diets were listed in Table 1.

**Table 1:** Ingredients and nutrient levels of the basal diets (as-fed basis)

Items	d 1 to 13	d 14 to 20	d 21 to 38
Ingredients, %			
Corn	24.86	26.04	20.24
Soybean meal	32.14	29.17	30.98
Wheat flour	15.00	15.00	15.00
Brown rice	15.00	15.00	15.00
Corn gluten	4.00	4.50	5.00
Fermented cottonseed protein	1.50	1.50	1.50
Hydrolyzed feather meal	1.00	1.00	1.50
Soybean oil	1.80	4.00	7.50
L-Lysine H_2_SO_4_	0.41	0.46	0.37
DL-Methionine	0.24	0.26	0.22
L-Threonine	0.10	0.12	0.09
Limestone	1.20	1.00	0.90
Monocalcium phosphate	0.95	0.57	0.42
NaCl	0.25	0.23	0.23
Choline chloride	0.10	0.10	0.10
Sodium humate	0.10	0.10	0.10
Phytase	0.02	0.02	0.02
Emulsifier	0.03	0.03	0.03
Montmorillonite	0.30	0.00	0.00
Premix^a^	1.00	0.90	0.80
Nutrient levels, % or indicated units			
Metabolic energy, MJ/kg^b^	12.00	12.55	13.30
Crude protein	24.14	23.37	23.30
Lysine	1.37	1.29	1.28
Methionine	0.35	0.33	0.38
Methionine + cystine	0.69	0.66	0.74
Calcium	0.89	0.74	0.69
Total phosphorus	0.67	0.57	0.54

^a^Premix provided (per kg of diet): vitamin A 9,000 IU, vitamin D_3_ 2,000 IU, vitamin E 11 IU, vitamin K_3_ 1.0 mg, vitamin B_1_ 1.2 mg, vitamin B_2_ 5.8 mg, vitamin B_6_ 2.6 mg, vitamin B_12_ 0.012 mg, niacin 66 mg, pantothenic acid 10 mg, biotin 0.10 mg, folic acid 0.7 mg, I 0.65 mg, Se 0.35 mg

^b^Calculated values

The broilers had free access to feed and water through individual feeders and drinkers in each cage. The experimental house was kept in 23 h of light and 1 h of darkness every day. The temperature was controlled at 34–35 °C in the first week, then decreased by 2℃ every week, and finally controlled between 24–26 °C. The relative humidity was 45%–55%. Thirty-six and 18 cages were randomly selected for the control and MA group, respectively. Then, the body weight (BW) was recorded at d 0, feeding phase switches (d 13/20), and slaughter (d 38) on the cage basis. Average daily gain (ADG) was calculated for the abovementioned feeding phases separately.

### Carcass traits and sample collection

Broilers were processed in a commercial slaughterhouse at d 38. One hundred broilers per group were selected according to the average BW of each group, electrically stunned, and killed by exsanguination. The ratio between carcass weight and live BW was the dressing percentage. The half-eviscerated weight and eviscerated weight were recorded to calculate the percentage of half-eviscerated yield and eviscerated yield. Then, about 6 g of left pectoralis major (PM) muscle was isolated and kept at 4 °C for drip loss measurement. Furthermore, a piece of left PM muscle (~ 20 g) was collected and stored at −80 °C for chemical composition analysis, RNA extraction, and untargeted metabolomics study.

### Drip loss

Fresh PM muscle samples (30 mm × 15 mm × 5 mm, ~ 6 g) were weighed (W1). The PM sample was hung by threading an iron wire through one end of muscle, and then placed into an air-filled plastic bag with no contact with the bag wall. It was hung in a refrigerator at 4 °C for 24 h. After that, muscle samples were dried and weighed again (W2) to calculate the drip loss, using the following equation: drip loss (%) = (W1 − W2)/W1 × 100.

### Chemical composition analysis

The routine nutritional composition of chicken meat was analyzed according to the recognized Association of Analytical Chemists methods [21]. Moisture content was assayed by the vacuum freeze-drying method for 72 h. Crude protein and intramuscular fat (IMF) levels were determined by the Kjeldahl and Soxhlet extraction methods, respectively. The determination of fatty acids and amino acids was described in our previous work [18].

### RNA extraction and real-time PCR analysis

Total RNA from PM muscle was extracted using RNAiso Plus (Takara, Dalian, China) and converted to cDNA by a PrimeScript^TM^ RT reagent kit (RR047A, Takara, China) according to the manufacturer’s instructions. SybrGreen-based quantitative PCR was carried out in a qTOWER 2.2 thermocycler (Analytik Jena, Jena, Germany). Primers specific for *GAPDH* mRNA and inflammation markers, including *IL8L1*, *IL1β*, *CCL4*, *CCL5*, *CCL19*, *CCR7*, and *iNOS* were designed by published GenBank sequence and were synthesized by Genewiz Co., Ltd. (Suzhou, China). *GAPDH* was used as the reference gene. The primers used in this study were presented in Table 2. Relative gene expression was calculated by 2^−ΔΔCt^ method [22].

**Table 2:** The primers for RT-qPCR assays

Target genes	Forward primers (5′→3′)	Reverse primers (5′→3′)
*IL8L1*	TGGCTCTTCTCCTGATCTCAATGG	CACTGGCATCGGAGTTCAATCG
*IL1β*	CCCTCCTCCAGCCAGAAAGTG	TGTAGCCCTTGATGCCCAGTG
*CCL4*	TCTCGCTCTTCTCATCGCATCC	CTGGTGCTGTAGTGCCTCTGG
*CCL5*	GCTGTGTCCCTCTCCATCCTC	AGTTGAAGCAGCACACGGTTG
*CCL19*	GTATGCTGGCAACAACGTCCTC	ATGAACACGGTGGCAGGGATG
*CCR7*	CGACGACTATGACGCCAACAC	ATCACGGACCTCCTTCTTCTCAC
*iNOS*	CCTGGAGGTCCTGGAAGAGT	CCTGGGTTTCAGAAGTGGC
*GAPDH*	GAAGCAGGACCCTTTGTTGG	TCTATCAGCCTCTCCCACCT

### Untargeted metabolomics study

Metabolite concentrations in PM muscle samples of broilers were quantified using UHPLC-MS/MS (Thermo Fisher Scientific, Wilmington, USA) in both positive and negative ionization modes. Fifty mg muscle samples were used. The metabolites were extracted using a 400 µL methanol:water (4:1, v/v) solution with 0.02 mg/mL L-2-chlorophenylalanine as internal standard. After centrifugation at 13,000 × *g* for 15 min at 4 °C, the supernatant was carefully transferred to sample vials for LC–MS/MS analysis. Two μL of the sample was separated by HSS T3 column (2.1 mm × 100 mm, 1.8 μm) at 40 °C, and the flow rate was 0.4 mL/min. Mobile phase A was 95% water and 5% acetonitrile (containing 0.1% formic acid). Mobile phase B was 47.5% acetonitrile, 47.5% isopropanol, and 5% water (containing 0.1% formic acid). The gradient was 0 B at 0 min, 24.5% B at 3.5 min, 65% B at 5 min, 100% B at 5.5 min, maintenance for 1.9 min, and a reduction to 0 B for 1.6 min, with a 1 min re-equilibration. A Q Exactive HF-X Mass Spectrometer equipped with an electrospray ionization source was used. The parameters were set as follows: heater temperature, 425 °C; capillary temperature, 325 °C; sheath gas flow rate, 50 arb; aux gas flow rate, 13 arb; spray voltage floating, −3,500 V in negative mode and 3,500 V in positive mode. Full MS resolution was 60,000, and MS/MS resolution was 7,500. Data acquisition was performed with the Data Dependent Acquisition mode. The detection was performed over a mass range of 70–1,050 *m/z*.

After data preprocessing and annotation by HMDB (http://www.hmdb.ca) and Metlin (https://metlin.scripps.edu) as described previously [23], the obtained data were applied for principal component analysis (PCA) and partial least squares discriminant analysis (PLS-DA). Metabolites with variable importance projection (VIP) > 1.0 and *P* < 0.05 were differential metabolites. To further interpret the biological significance of metabolites, metabolic pathway analysis was performed based on the KEGG database (http://www.genome.jp/kegg).

### RNA extraction, sequencing and analysis

According to the average drip loss, 6 PM samples in each group were selected for total RNA extraction using TRIzol^®^ reagent according to the manufacturer’s instructions. The RNA quality was determined by an Agilent 5300 Bioanalyzer (Agilent Technologies, CA, USA). RNA purification, reverse transcription, library construction, and sequencing were performed at Shanghai Majorbio Bio-pharm Biotechnology Co., Ltd. (Shanghai, China) according to the manufacturer’s instructions. Then, the libraries were sequenced with the NovaSeq 6000 sequencer (2 × 150 bp read length). The fastp (version 0.18.0) was used to obtain high-quality, clean reads. The rRNA-mapped reads were removed by using the Bowtie2 (version 2.2.8) tool. The software Stringtie and HISAT2 were used to align the clean reads to the reference genome. DESeq2 software was used for differential expression analysis between two groups, and the genes with *P* < 0.05 and an absolute fold change > 2.0 were considered differentially expressed. Then differentially expressed genes (DEGs) were carried out on the KEGG pathway enrichment analysis by KOBAS [24]. The *P*-value (*P* < 0.05) was used to determine the significantly enriched KEGG pathways. All identified genes were loaded into Gene Set Enrichment Analysis (GSEA) to identify enriched biological processes by comparing them against KEGG gene sets on the Majorbio I-Sanger Cloud Platform (https://cloud.majorbio.com).

### Statistical analysis

Data were presented as mean ± SEM, and analyzed by the unpaired two-tailed Student’s *t*-test procedures of SAS (v.9.2, SAS Institute, USA). For growth performance data, each cage was treated as the experimental replicate. Other indices were analyzed by one broiler as a replicate. The value of *P* < 0.05 was considered significant.

## Results

### Growth performance and carcass traits

**Fig. 1:**
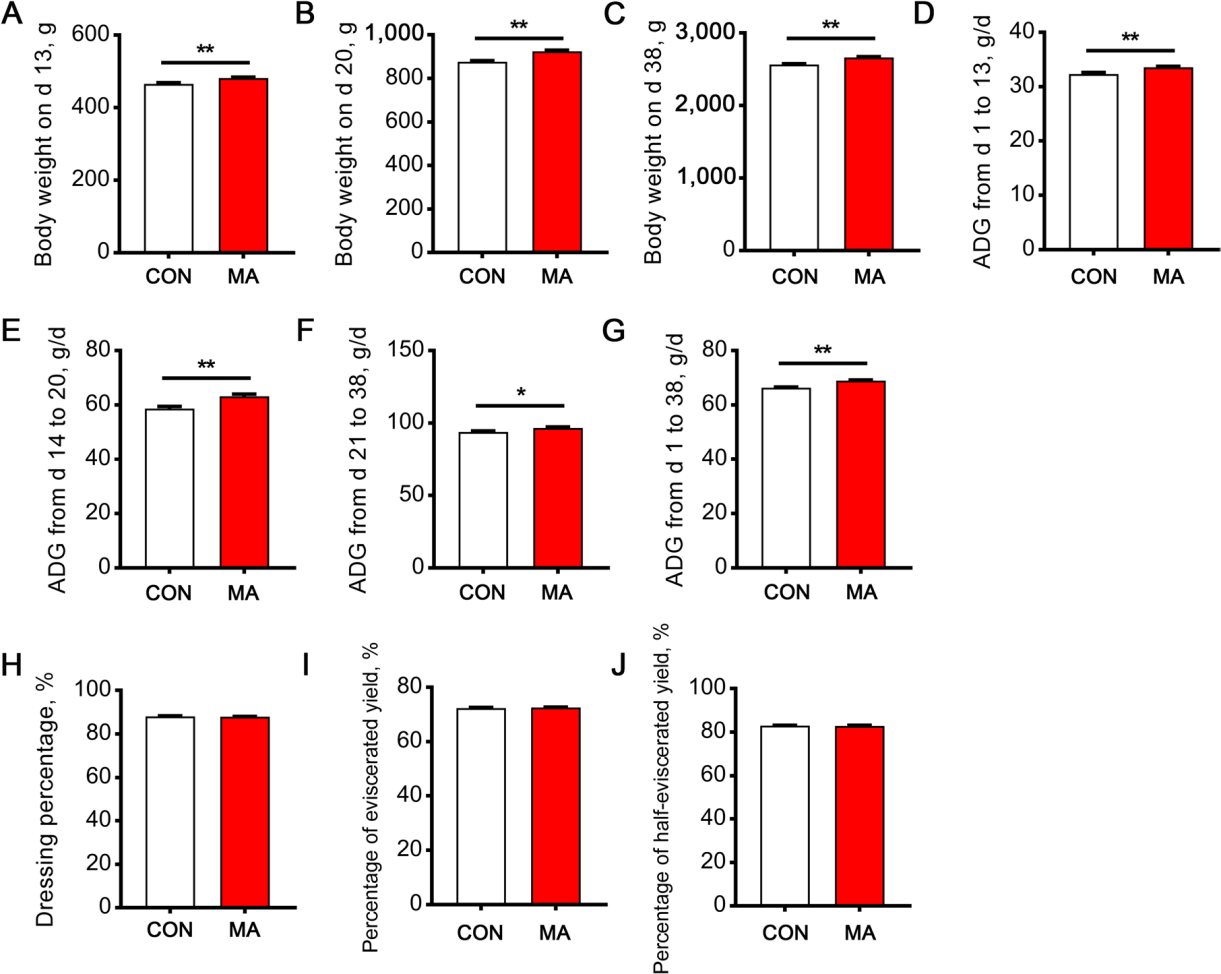
Effects of dietary MA supplementation on growth performance and carcass traits of broilers. **A–C** The body weight at d 13, 20, and 38. **D–G** The average daily gain from d 1 to 13, d 14 to 20, d 21 to 38, and d 1 to 38. **H** Dressing percentage. **I** Percentage of eviscerated yield. **J** Percentage of half-eviscerated yield. Data are expressed as the mean ± SEM. **A–G**: *n* = 36 for the control group and *n* = 18 for the MA group. **H–J**: *n* = 100. Statistics were performed with Student’s *t*-test. ^*^P < 0.05, ^**^P < 0.01

As shown in Fig. 1, dietary supplementation of MA significantly increased the BW of broilers at d 13, 20, and 38 compared with the control group (*P* < 0.01, Fig. 1A–C). From d 1 to 13, d 14 to 20, d 21 to 38, and d 1 to 38, the ADG of broilers in the MA group was all increased (*P* < 0.05, Fig. 1D–G). In addition, MA supplementation had no effects on carcass traits, including dressing percentage, percentage of eviscerated yield, and percentage of half-eviscerated yield (Fig. 1H–J).

### Drip loss

**Fig. 2:**
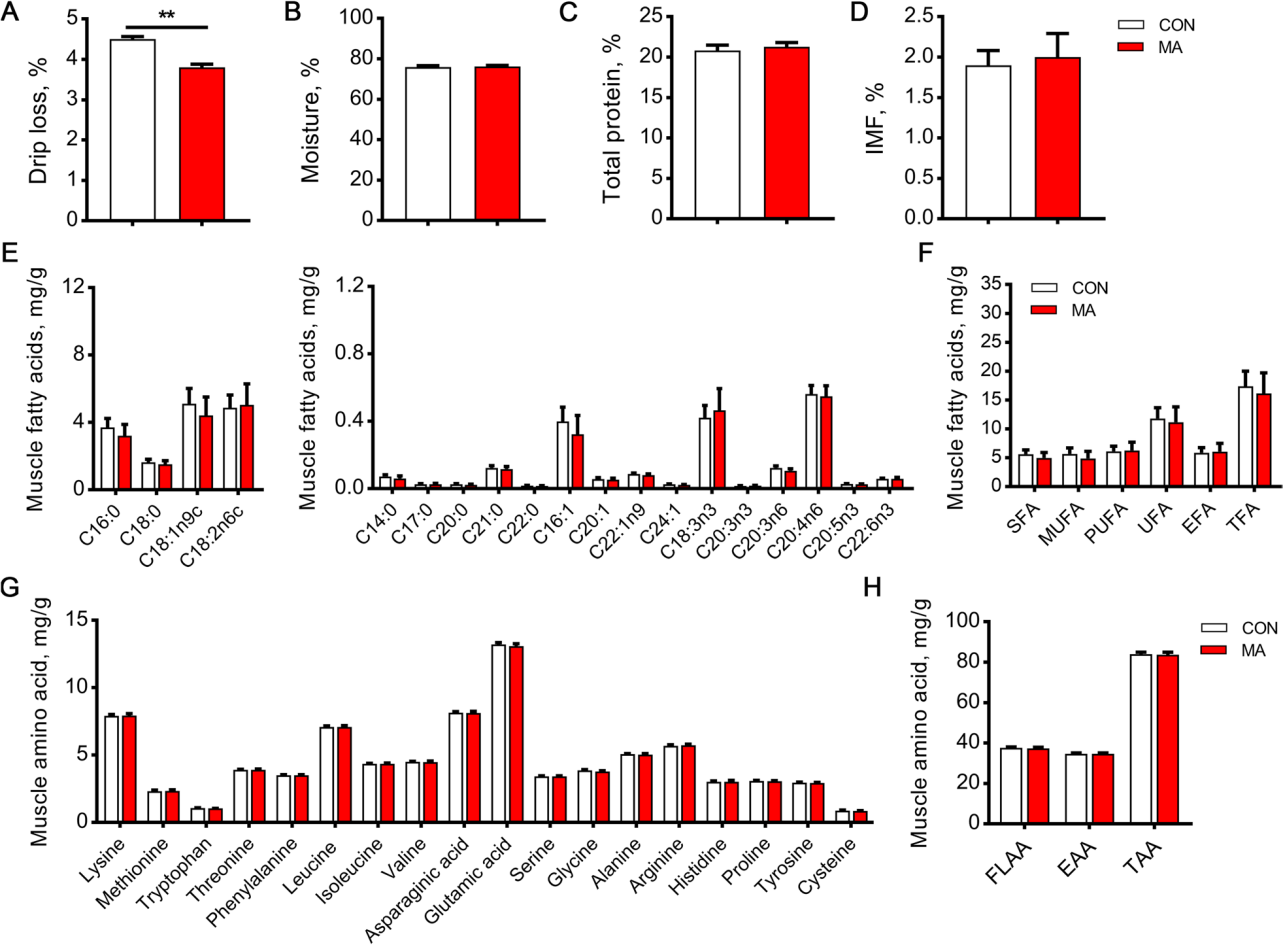
Effects of dietary MA supplementation on meat quality and chemical composition of broilers. **A** Drip loss was measured at 24 h after slaughter. **B** Moisture. **C** Total protein. **D** Intramuscular fat. **E–F** Muscle fatty acids composition. SFA, saturated fatty acids; MUFA, monounsaturated fatty acids; PUFA, polyunsaturated fatty acids; UFA, unsaturated fatty acids; EFA, essential fatty acids; TFA, total fatty acids. **G–H** Muscle amino acids composition. FLAA, flavor amino acids (Asp, Glu, Ser, Gly, Ala, Thr). EAA, essential amino acids (Lys, Met, Trp, Thr, Phe, Leu, Ile, Val). TAA, total amino acids. Data are expressed as the mean ± SEM. **A**: *n* = 100; **B–H**: *n* = 6. Statistics were performed with Student’s *t*-test. ^**^P < 0.01

Compared with the broilers in the control group, dietary supplementation of MA significantly decreased the drip loss of PM muscle (*P* < 0.01, Fig. 2A), while did not affect the contents of moisture, protein, and IMF (Fig. 2B–D). Similarly, MA also did not affect the composition of fatty acids (Fig. 2E and F) and amino acids (Fig. 2G and H) of PM muscle.

### Muscle metabolic profiles

**Fig. 3:**
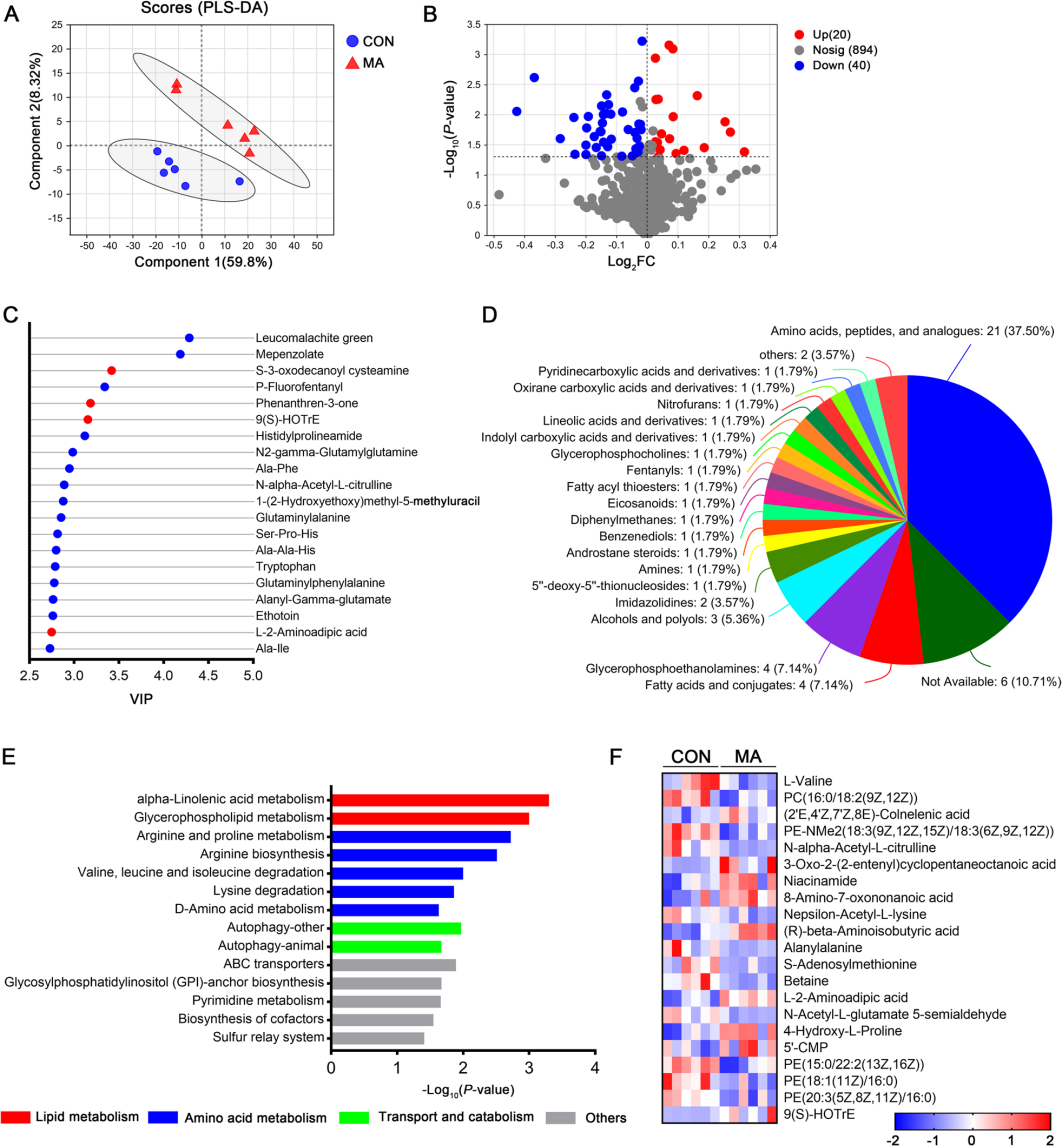
Effects of dietary MA supplementation on PM muscle metabolome in broilers (*n* = 6). **A** Partial least squares discriminant analysis (PLS-DA) shows the separation of data depending on MA supplementation. **B** Volcano plots of all metabolites. Red modes and blue modes represent up-regulated and down-regulated metabolites in the MA group, respectively. The dotted horizontal line indicates the threshold for a *P*-value of 0.05. **C** VIP scores show the top 20 metabolites responsible for the data separation. Red modes and blue modes represent up-regulated and down-regulated metabolites in the MA group, respectively. **D** The classification of total differential metabolites based on HMDB database annotation. **E** Enrichment analysis of differential metabolites based on the KEGG database. **F** The heatmap showing differential metabolites enriched in KEGG pathways

Untargeted metabolomics approaches were performed to measure the metabolic profiles of PM muscle. PLS-DA exhibited a clear separation between control and MA groups (Fig. 3A). A total of 954 metabolites were identified. Among them, 20 metabolites were up-regulated, while 40 were down-regulated in the MA group (Fig. 3B). The top 20 metabolites responsible for the greatest separation of the data were also revealed using VIP scores (Fig. 3C). According to VIP scores, leucomalachite green, mepenzolate, *p*-fluorofentanyl, histidylprolineamide, and N2-gamma-glutamylglutamine were top five down-regulated metabolites (blue nodes in Fig. 3C). S-3-Oxodecanoyl cysteamine, phenanthren-3-one, 9(S)-HOTrE, and L-2-aminoadipic acid (2-AA) were top four up-regulated metabolites (red nodes in Fig. 3C). Interestingly, among these 60 differential metabolites, 21 were amino acids, peptides, and analogues, 4 were fatty acids and conjugates, and 4 were glycerophosphoethanolamines (Fig. 3D). The relative abundances of differential metabolites were listed in Table 3, which demonstrated that the abundances of four metabolites belonging to amino acids, peptides, and analogues were increased in the MA group, such as 3-aminoisobutanoic acid, β-aminoisobutyric acid (BAIBA), 2-AA, and 4-hydroxy-L-proline. Additionally, differential metabolites involved in fatty acids and conjugates were all up-regulated in the MA group, including colnelenic acid, octadecanoic acid, oxononanoic acid, and eicosapentaenoic acid (EPA) (Table 3).

**Table 3:** List of differential metabolites in the PM muscle from broilers

Metabolites	VIP score	Fold change (MA/CON^a^)
Amino acids, peptides, and analogues		
3-Aminoisobutanoic acid	2.07	1.78
L-Alanyl-L-valine	2.29	0.42
L-Valine	1.28	0.71
N-alpha-Acetyl-L-citrulline	2.89	0.23
Alanyltryptophan	2.48	0.27
Ala-ILE	2.73	0.23
Nepsilon-Acetyl-L-lysine	1.78	0.55
(R)-beta-Aminoisobutyric acid	2.28	1.99
Alanylalanine	2.66	0.18
N2-gamma-Glutamylglutamine	2.98	0.29
Betaine	1.74	0.60
L-2-Aminoadipic acid	2.75	2.68
N-Acetyl-L-glutamate 5-semialdehyde	2.65	0.29
4-Hydroxy-L-proline	1.20	1.22
Histidylprolineamide	3.12	0.10
Glutaminylphenylalanine	2.78	0.21
Phenylalanyl-gamma-glutamate	2.39	0.27
Ala-phe	2.95	0.19
Glutaminylalanine	2.85	0.21
Alanyl-gamma-glutamate	2.76	0.25
2-Amino-4-[(2-hydroxy-1-oxopropyl)amino]butanoic acid	1.89	0.53
Fatty acids and conjugates		
(2'E,4'Z,7'Z,8E)-Colnelenic acid	1.04	1.22
9,10-Epoxy-18-hydroxy-octadecanoic acid	1.96	2.10
8-Amino-7-oxononanoic acid	1.57	1.42
Eicosapentaenoic acid	1.52	1.56
Glycerophosphoethanolamines		
PE-NMe2(18:3(9Z,12Z,15Z)/18:3(6Z,9Z,12Z))	1.18	0.79
PE(15:0/22:2(13Z,16Z))	1.04	0.78
PE(18:1(11Z)/16:0)	1.26	0.73
PE(20:3(5Z,8Z,11Z)/16:0)	1.40	0.68
Others		
Cyclopentanol	1.95	2.14
(2R,3R)-(-)-2,3-Butanediol	1.15	1.20
Lichenin	1.08	1.27
Ethotoin	2.76	0.18
S-4-Hydroxymephenytoin	2.57	0.24
S-Adenosylmethionine	1.31	0.67
Gaboxadol	1.42	1.45
Phenanthren-3-one	3.18	3.58
Isoetharine	2.03	1.97
Mepenzolate	4.19	0.03
Cyclopentaneoctanoic acid	2.48	4.48
S-3-oxodecanoyl cysteamine	3.42	4.62
*p*-Fluorofentanyl	3.34	0.02
PC(16:0/18:2(9Z,12Z))	1.21	0.75
(±)-Tryptophan	2.79	0.21
9(S)-HOTrE	3.15	15.85
{[5-(5-Nitro-2-furyl)-1,3,4-oxadiazol-2-YL]thio}acetic acid	1.11	0.77
Glycidamide	1.36	0.67
Niacinamide	1.41	1.41
5'-CMP	1.40	1.37
1-(2-Hydroxyethoxy)methyl-5-methyluracil	2.88	0.21
3,4-Dihydroxyhydrocinnamic acid	2.19	0.35
Leucomalachite green	4.29	0.02
PC(P-16:0/20:5(5Z,8Z,10E,14Z,17Z)-OH(12))	1.33	0.68
(±)-Pelletierine	2.05	2.14
PS(5-iso PGF2VI/22:0)	1.21	0.73
PE(22:0/18:1(12Z)-2OH(9,10))	1.31	0.73

^a^*CON* Control group, *MA* L-malic acid

We identified 14 KEGG pathways enriched with differential metabolites (*P* < 0.05, Fig. 3E). Metabolites involved in these pathways were exhibited by heat map (Fig. 3F). Following the dramatically changed profiles of metabolites relating to amino acids and fatty acids, seven KEGG pathways were enriched, including “alpha-linolenic acid metabolism”, “glycerophospholipid metabolism”, “arginine and proline metabolism”, “arginine biosynthesis”, “valine, leucine, and isoleucine degradation”, “lysine degradation”, and “D-amino acid metabolism” (*P* < 0.05, Fig. 3E). These results highlighted the importance of lipid and amino acid metabolism behind the beneficial effects of MA on drip loss.

### RNA sequencing and differential expression analysis

**Fig. 4:**
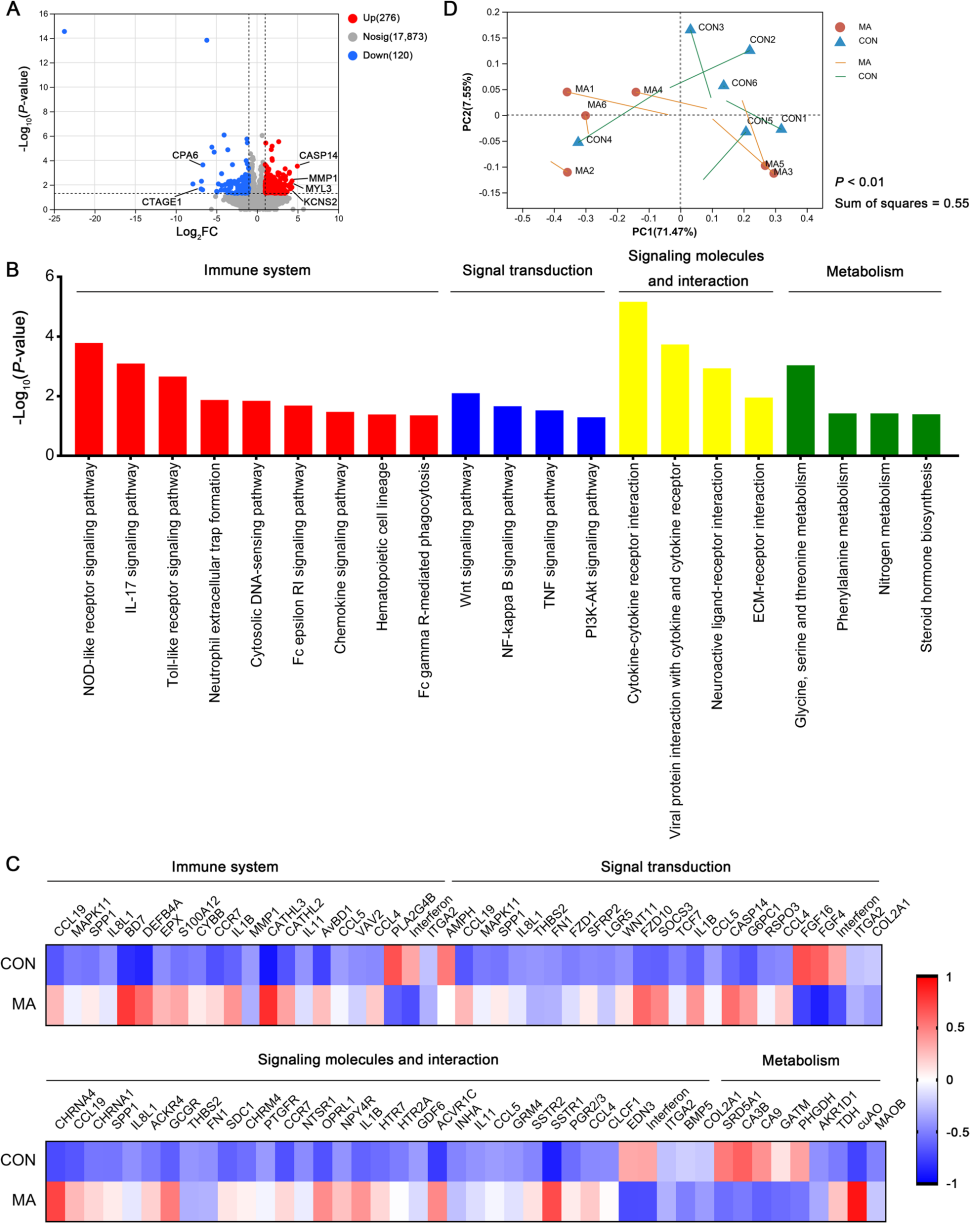
Gene expression differences of PM muscle between treatments were measured by RNA-seq (*n* = 6). **A** Volcano plot of differentially expressed genes (DEGs). **B** KEGG analysis of DEGs. **C** Heatmap for the expression profile of DEGs enriched in KEGG pathways as listed in B. **D** Procrustes analysis between transcriptome and metabolome of PM muscle

Compared with the control group, 396 DEGs were identified in PM muscle from the MA group, including 276 up-regulated genes and 120 down-regulated genes (Fig. 4A). KEGG pathway analysis of DEGs revealed significant enrichment of genes associated with immune system, signal transduction, signaling molecules and interaction, and metabolism (Fig. 4B). Briefly, NOD-like receptor signaling pathway, IL-17 signaling pathway, and Toll-like receptor signaling pathway were the three most enriched immune system pathways. The top three enriched signal transduction pathways were Wnt signaling pathway, NF-κB signaling pathway, and TNF signaling pathway. The top three most enriched signaling molecules and interaction pathways were cytokine-cytokine receptor interaction, viral protein interaction with cytokine and cytokine receptor, and neuroactive ligand-receptor interaction. Furthermore, the KEGG annotation results revealed that glycine, serine and threonine metabolism, phenylalanine metabolism, and nitrogen metabolism were the three most enriched metabolic pathways (Fig. 4B). As displayed by the heat map, most DEGs involved in the immune system, signal transduction, and signaling molecules and interaction were up-regulated in the MA group (Fig. 4C). In this study, Procrustes analysis was also performed, and a significant relatedness (sum of squares = 0.55, *P* = 0.006) between transcriptome and metabolome of PM muscle was observed (Fig. 4D).

### Immune response in the skeletal muscle

**Fig. 5:**
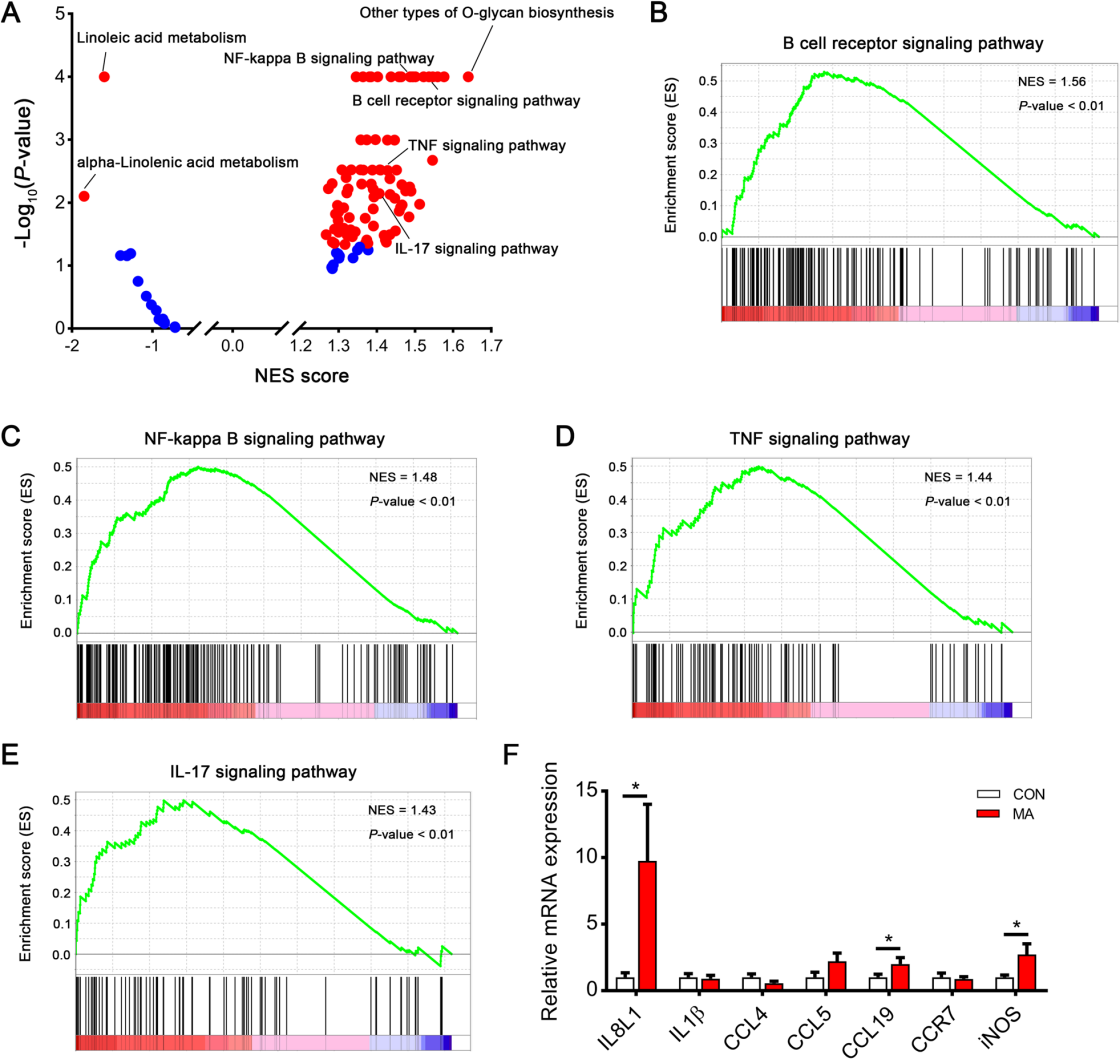
Effects of dietary MA supplementation on immune response in PM muscle. **A** GSEA was performed to analyze the pathways affected by MA supplementation in PM muscle. Significant biological pathways are labeled in red. **B** GSEA in B cell receptor signaling pathway. **C** GSEA in NF-κB signaling pathway. **D** GSEA in TNF signaling pathway. **E** GSEA in IL-17 signaling pathway. **F** Relative mRNA expression levels of inflammatory markers in PM muscle. Data are expressed as the mean ± SEM. *n* = 6. Statistics were performed with Student’s *t*-test. ^*^P < 0.05

To further examine the effect of MA on PM muscle at the molecular level, GSEA was performed to analyze the pathways affected by dietary MA supplementation. GSEA analysis revealed some enhanced immune response pathways, such as B cell receptor signaling pathway, NF-κB signaling pathway, TNF signaling pathway, and IL-17 signaling pathway (Fig. 5A–E). Additionally, α-linolenic acid metabolism and linoleic acid metabolism were down-regulated in the MA group (Fig. 5A). Consistently, real-time PCR confirmed that inflammation markers such as *IL8L1*, *CCL19*, and *iNOS* were significantly increased in the PM muscle from the MA group (*P* < 0.05, Fig. 5F).

## Discussion

Meat WHC is the most important functional characteristic of fresh meat, which is associated with meat color, tenderness, juiciness, nutritive value, and shelf life, and can be reflected by drip loss. Higher drip loss would affect consumer’s purchase intention and result in severe economic loss in the meat industry. Paralleling an increase in drip loss, some soluble constituents, including vitamins, minerals, and amino acids will be taken away. Sensory quality attributes of meat will also be deteriorated, such as juiciness, tenderness, and flavor intensity [25, 26]. In the current study, dietary 0.8% MA supplementation increased the growth performance and decreased the drip loss of broilers at large-scale rearing. These results were in agreement with our previous study where the ADG, feed conversion ratio, and WHC were all significantly improved by dietary MA in broilers [19].

Metabolomics has been successfully used as an effective method to describe the characteristics of chicken meat after specific dietary treatment [27, 28]. In this study, the metabolome of breast muscle identified 60 differential metabolites. Among them, four typical metabolites including 2-AA, BAIBA, EPA, and nicotinamide were generally identified as key biomarkers for meat quality and growth performance evaluation. 2-AA is a metabolite of lysine and has been reported to suppress myofibrillar protein degradation in C2C12 myotubes [29]. An increased content of 2-AA was also observed in the breast muscle of the duck during skeletal muscle development [30]. Besides, as a metabolite of valine, BAIBA could abrogate the LPS-induced secretion of pro-inflammatory cytokines in 3T3-L1 cells [31]. Thus, the improved growth performance in the present study was probably due to the increased 2-AA and BAIBA contents in the MA group. Omega-3 long-chain EPA has been extensively reviewed to expert benefits throughout life, including use during pregnancy for normal fetal development, reduction of many cardiovascular diseases, and potential uses in Alzheimer’s disease [32], indicating that high levels of EPA in the MA group may enhance the beneficial effect of chicken meat. Moreover, increased nicotinamide level was also observed in the breast muscle from the MA group. Nicotinamide plays a critical role in antioxidant activity through the activation of Foxo3a in osteoblasts and human gestational tissues [33, 34]. It has been reported that dietary supplementation of 50 or 100 mg/kg nicotinamide could reduce drip loss and lightness of chicken meat by reducing the expression of protein ubiquitin degradation genes *FBXO9* and *FBXO32* [35], indicating the high levels of nicotinamide in the MA group has an important impact on WHC of meat. In conclusion, the meat metabolome provided solid evidence regarding MA’s improved growth performance and WHC of broilers with identified metabolites. Metabolic pathway analysis further identified that lipid and amino acid metabolism was changed in the breast muscle by MA treatment. This finding was supported by our previous study where surplus dietary isoleucine intake decreased drip loss of finishing pigs [36]. The coordination of valine and isoleucine on WHC was also reported in finishing pigs [37]. Ma et al. [38] demonstrated that dietary 1% arginine supplementation decreased drip loss and increased IMF content of finishing pigs, however, increased digestible arginine:lysine ratio had no significant effect on meat quality in broilers [28]. The effects of arginine on chicken meat quality may require further investigation. Consistently, based on the KEGG enrichment analysis and DEGs, two amino acid metabolism pathways were also identified, including phenylalanine metabolism, and glycine, serine and threonine metabolism. Therefore, these alterations of amino acid metabolism induced by MA treatment may be the cause of the improvements in meat WHC.

In this study, we analyzed the global gene expression in breast muscle samples from the control and MA groups by RNA-seq. KEGG enrichment analysis revealed that nine pathways involved in the immune system were significantly enriched based on 396 DEGs. Consistently, GSEA analysis demonstrated that B cell receptor signaling pathway, NF-κB signaling pathway, TNF signaling pathway, and IL-17 signaling pathway were enhanced in the MA group. The NF-κB transcription factor is a key regulator of the immune system and can be activated by inflammatory signaling [39], which was consistent with the up-regulation of *I8L1*, *CCL19*, and *iNOS* in the MA group. H_2_O_2_-induced oxidative stress was reported to induce apoptosis and abnormal autophagy, and impair WHC and shear force by inhibiting the activation of NF-κB signaling pathway in the thigh and breast muscle of broilers [40, 41]. Dietary taurine could improve growth performance, pH value, and tenderness of broilers challenged with H_2_O_2_ by activating NF-κB signaling [42]. These findings are in agreement with a recent work reporting that the NF-κB pathway was enhanced in wooden breasts [43], which was characterized by hardened areas, pale, and ridge-like bulges at the caudal end. Strikingly, WHC gradually decreased concomitant with the worsened wooden breast myopathy [43]. Consequently, proper activation of the NF-κB signaling pathway is important for chicken meat quality. The host-protective capacity of IL-17 signaling was also reported. IL-17 is an inflammatory cytokine mainly produced by type 17 T helper (Th17) cells, but its role in regulating meat quality remains unclear. IL-17-mediated inflammation is crucial for microbial clearance, whereas unrestricted IL-17 signaling leads to immunopathology [44]. For example, activation of IL-17 signaling was found in broiler lungs in pathogen-induced respiratory disease [45]. In the future, the crosstalk between immune cells and myoblasts could be studied to further highlight the importance of immune response in regulating meat WHC.

## Conclusions

In conclusion, the present study demonstrated that the growth performance and meat drip loss were notably improved by dietary MA supplementation under commercial conditions. Meat metabolome and transcriptome identified key metabolites and amino acid metabolism connecting to the improvement of drip loss. Importantly, a proper immune response was necessary for chicken meat quality. The present study introduces us an effective feed additive to improve WHC in poultry production, and provides us with a new approach to control the meat quality from farm to table.

## Data Availability

The data analyzed during the current study are available from the corresponding author on reasonable request.

## Abbreviations

2-AA: L-2-aminoadipic acid
AA: Arbor Acres
ADG: Average daily gain
BAIBA: β-Aminoisobutyric acid
BW: Body weight
DEGs: Differentially expressed genes
EPA: Eicosapentaenoic acid
GSEA: Gene set enrichment analysis
IMF: Intramuscular fat
MA: L-malic acid
PCA: Principal component analysis
PLS-DA: Partial least squares discriminant analysis
PM: Pectoralis major
PSE: Pale, soft, and exudative
Th17: Type 17 T helper
VIP: Variable importance projection
WHC: Water-holding capacity
